# Experiencing El Niño conditions during early life reduces recruiting probabilities but not adult survival

**DOI:** 10.1098/rsos.170076

**Published:** 2018-01-17

**Authors:** Sergio Ancona, J. Jaime Zúñiga-Vega, Cristina Rodríguez, Hugh Drummond

**Affiliations:** 1 Centro Tlaxcala de Biología de la Conducta, Universidad Autónoma de Tlaxcala, Tlaxcala, México; 2 Departamento de Ecología y Recursos Naturales, Facultad de Ciencias, Universidad Nacional Autónoma de México, Ciudad de México, México; 3 Departamento de Ecología Evolutiva, Instituto de Ecología, Universidad Nacional Autónoma de México, Ciudad de México, México

**Keywords:** early-life stress, life history, survival, recruitment, capture–recapture, El Niño Southern Oscillation

## Abstract

In wild long-lived animals, analysis of impacts of stressful natal conditions on adult performance has rarely embraced the entire age span, and the possibility that costs are expressed late in life has seldom been examined. Using 26 years of data from 8541 fledglings and 1310 adults of the blue-footed booby (*Sula nebouxii*), a marine bird that can live up to 23 years, we tested whether experiencing the warm waters and food scarcity associated with El Niño in the natal year reduces recruitment or survival over the adult lifetime. Warm water in the natal year reduced the probability of recruiting; each additional degree (°C) of water temperature meant a reduction of roughly 50% in fledglings' probability of returning to the natal colony as breeders. Warm water in the current year impacted adult survival, with greater effect at the oldest ages than during early adulthood. However, warm water in the natal year did not affect survival at any age over the adult lifespan. A previous study showed that early recruitment and widely spaced breeding allow boobies that experience warm waters in the natal year to achieve normal fledgling production over the first 10 years; our results now show that this reproductive effort incurs no survival penalty, not even late in life. This pattern is additional evidence of buffering against stressful natal conditions via life-history adjustments.

## 1. Introduction

Early-life exposure to stressful conditions may have short- or long-term fitness consequences [1,2]. Food shortage, disease, extreme weather or high population density during pre- or postnatal development may translate into reduced birthweight, growth or pre-breeding survival [1,3]. Developing offspring can sometimes get over a poor start in life, yet pay delayed costs of initial setbacks or compensatory responses triggered by poor early-life conditions [4]. Delayed costs of early-life stresses can be expressed as shortened longevity or reduced lifetime reproductive success [5,6], or accelerated senescence [7]. Nonetheless, recent long-term studies show that in nature early-life stresses can also elicit changes in life-history traits that allow organisms to mitigate or neutralize their potential impacts on fitness [8,9].

Whether we detect vulnerability or resilience to early-life stresses may depend on the nature of the stress (i.e. origin, magnitude, timing and duration) but also on the ability of studies to estimate impacts on diverse fitness components over the lifespan [2,9]. Impacts of early-life stresses after sexual maturity, however, are still seldom explored [7,10,11], and because the evidence for delayed costs of poor start in life comes mainly from relatively short-term experiments in captivity, we know little about how pervasive and important these impacts are in nature [9,12].

Potentially, early-life stresses may give rise to differences among cohorts in recruitment and adult survival, and translate into demographic changes [1,10,13,14]. Survival costs of a poor start in life during early adulthood have been extensively documented by experiments using captive members of short-lived species [15–18]. Comparable impacts have been examined only occasionally in long-lived species under natural conditions [7,11,19], and the general expectation that natural selection favours costs being expressed late in life (when expression is less likely) needs to be evaluated more extensively in natural populations [20]. Additionally, our knowledge of how early-life environments influence recruitment rates in nature is particularly limited [21–23].

Using the blue-footed booby (*Sula nebouxii*) as a model organism, we tested whether food shortage and other challenges associated with El Niño conditions during the natal year (spanning from prenatal to early post-fledging development) translate into impaired recruitment and increased mortality in the adult stage up to an age of 23 years. Delayed costs arising from impacts of El Niño during early development have been tested in only two species. In the black-browed albatross (*Thalassarche melanophrys*), immature survival declined but timing of recruitment was unaffected [24]. In our study species, birds recruited earlier and avoided costs on longevity and breeding success over at least the first 10 years of life by means of other life-history adjustments [25]. Nonetheless, whether costs of natal El Niño conditions are expressed as reductions in recruitment and adult survival, and whether these costs are evident only much later in life (after 10 years of age) remain as open questions. We expected relatively low recruitment and adult survival of boobies that experienced warm water conditions associated with El Niño during the year when they were born and fledged. Such long-term developmental impacts of El Niño have the potential to influence population dynamics [25,26], and their appraisal may provide important insights into the ability of wild populations to respond to major climatic changes [2,9].

## 2. Material and methods

### 2.1. Study system

We used data spanning 26 years (1988–2013) from a colony of blue-footed boobies on Isla Isabel, Mexico. This insular population is located in the eastern tropical Pacific and sustains itself largely on the anchovies and herrings of the surrounding oceanic waters [27]. Nestling growth, recruitment, adult survival and reproduction in this population decrease when local ocean warming in the course of an El Niño event depletes local productivity and prey availability [28,29]. Male and female boobies generally recruit at age 2–6 years [30,31] and can live 20 years or more [32]. Their breeding success increases gradually up to age 8–11 years and declines progressively thereafter [33,34], although they can recover from age-related decay by skipping breeding events [35].

Every year from 1988 to 2013, all fledglings were ringed and resighted as breeders (with clutch or brood) in the course of systematic censuses every 3–6 days during five months (February–July) in a fixed study area (26 889 m^2^) [30]. We based our analyses exclusively on resightings during two months (April–May) to meet the assumption of capture–recapture analyses that the time span between capture occasions (e.g. 12 months) must be substantially longer than the duration of the capture events (e.g. 2 months) [36]. During April–May, a representative sample of fledglings that are ringed and recorded as breeders annually can be obtained [37].

Our initial sample consisted of 8541 fledglings from 15 cohorts (1988–2009 except 1990, 1992, 1993, 1995, 1996, 2003 and 2005 from which no chicks fledged or when no fledglings were banded). For this initial sample, we scored recruitment (i.e. whether an individual fledgling was resighted in the natal colony with a partner and clutch during the first 6 years of life or never resighted again; 1–0 variable). The sex of fledglings was unknown. From this initial sample, we obtained a second sample including individual capture–recapture histories of 1310 fledglings (687 males and 623 females) that recruited at ages 2–6 years. These 1310 individuals were sexed by voice when they recruited (females grunt, males whistle) and accounted for 75.68% of recruits. We used this second sample to test whether El Niño-related conditions experienced in the natal year negatively affect adult survival.

In our second sample, average (±s.e.) recruiting ages were 4.77 ± 0.04 and 4.51 ± 0.05 years for males and females, respectively. Fledglings that were recorded as breeders for the first time either at age 1 year (*n* = 8) or 7–16 years (*n* = 412) were excluded from the analysis in order to reduce bias in demographic estimates: most 1-year-old recruits were recorded as breeders only once in 26 years, making it impossible to attribute their early disappearance to either mortality or emigration; whereas 7- to 16-year-old recruits may have bred earlier in life but escaped sampling [31]. Estimation of recruitment and survival probabilities in this colony may suffer from a little bias associated with temporal emigration [28,31] because Isla Isabel boobies rarely disperse from their natal colony [38] and are permanently faithful to their first breeding sites [39].

Oceanographic conditions experienced by boobies in early life were indexed by the average sea surface temperature anomaly (SSTA, °C) in the waters surrounding Isla Isabel during the natal calendar year. Positive values of SSTA denote warm El Niño conditions and potential reductions in food quality and quantity; negative values denote cold La Niña conditions [27,40]. We obtained monthly average values of SSTA at 21.5° N, 105.5° W (55 km southeast of Isla Isabel, the closest station) from the International Research Institute for Climate and Society (http://iri.columbia.edu/ website. Available: https://iridl.ldeo.columbia.edu/SOURCES/.NOAA/.NCEP/.EMC/.CMB/.GLOBAL/.Reyn_SmithOIv2/.monthly/.ssta/).

### 2.2. Recruitment analysis

We tested whether recruitment probabilities of booby fledglings (*n* = 8541) are associated with the average SSTA they experienced in their natal years using generalized linear mixed models (GLMMs) with binomial error distribution and a logit link function. We fitted four competing models to our data, including an intercept-only model (i.e. null model), a model considering fledglings' recruitment probabilities as a function of the number of years of monitoring, a model considering the potential impact of SSTA during the natal year (SSTAn) on fledglings' recruitment probabilities, and a model considering both the number of years of monitoring and SSTAn as covariates. In all these models, we included cohort as a random effect to account for unmeasured environmental conditions in the natal years (e.g. parasitism or population density).

We selected the model with the best fit to our data using the Akaike information criterion adjusted for small sample sizes (AIC_c_); the best fit was given by the model with the lowest AIC_c_ score. A difference between any particular model *i* and the best fitting model larger than 2 units in their AIC_c_ scores (Δ*_i_* > 2) was considered as a real difference in their fit to the data [41]. To reduce uncertainty in the process of model selection, we calculated Akaike weights (*w_i_*) as estimates of the relative support for each competing model and used a multi-model averaging approach [42] for a subset of the best fitting models which, together, had a cumulative *w*_i_ of approximately 0.95. These models were fitted using R (v. 3.1.3 [43]).

### 2.3. Capture–recapture analysis

To test whether SSTA experienced in the natal year influences adult survival, we used a capture–recapture analysis. We analysed the 26-year mark–recapture data by means of age-dependent models [44] implemented in the program MARK [45]. Individuals were classified into 14 age classes. The first age class included fledglings as well as 1-, 2- and 3-year-old individuals. We pooled these ages into a single (pre-adult) category because we focused our capture–recapture on adults, and most Isla Isabel boobies can be considered as reproductively mature adults from age 4, the average age of first breeding [30]. The next 12 age categories consisted of individuals from 4 to 15 years old, with each of these ages constituting one separate age class. Finally, in the last age class we included all individuals that were 16 years or older because sample sizes for each of these ages were too small to allow for age-specific parameter estimation. These 14 age classes adequately describe the variation in the survival rates of our colony of blue-footed boobies because the full age-dependent model, in which we estimated different age-specific survival probabilities for individuals of ages 16–23, had notably weaker support in our data (ΔAIC_c_ = 11.3 with respect to our model with 14 age classes). This result indicates little evidence of statistical differences in the survival rates of individuals between 16 and 23 years old, which is also evidenced by a general decline in the number of breeding individuals over the age of 16 years [32].

We used a maximum-likelihood procedure and a multi-model inference framework to estimate two parameters: apparent survival (*φ*) and recapture rate (*p*), as well as to assess whether natal conditions (estimated as the SSTA during the natal year) affect survival during adulthood. To avoid fitting an unnecessarily large number of models to our data, we searched first for the best parametrization for the recapture rate (*p*) following the procedure recommended by Lebreton *et al*. [44] and Doherty *et al*. [46]. This procedure consisted of testing different sources of variation for *p* (sex, year, and their additive and interactive effects) while maintaining the survival parameter varying across years, age categories and sexes. We did not test for differences in *p* among age categories to avoid increasing the total number of parameters to be estimated from our data. Thus, we assumed that most of the variation in recapture probability was caused by differences among years in the number of adult birds that were resighted rather than by differences in *p* among birds of different ages. Two of these models that examined variation in *p* had strong support in our data. The top model included only the effect of year, whereas the second model included an additive effect of year and sex. These two models had similar support in our data (ΔAIC_c_ = 1.4). However, because adding the effect of sex did not substantially improve model fit, in all the competing models described below we used inter-annual variation in *p* without distinguishing between males and females. Estimates of these recapture parameters can be found in electronic supplementary material, figure S1.

We built competing linear models in MARK to test the potential impact of SSTA during the natal year (SSTAn) on the survival probabilities of adults of all age categories. In addition, we considered the possibility that a potential interaction between natal (SSTAn) and current conditions (SSTA during the current year = SSTAc) affected adult survival. In all cases, we tested these hypotheses considering potential differences between sexes and age categories. Thus, we considered models in which *φ* varies as a function of age, sex, SSTAn, SSTAc, and additive and interactive effects of all these variables. In total, we fitted 27 competing models to our mark–recapture data, including an intercept-only model (i.e. a null model). Given that this analysis only embraced individuals recaptured as recruits at least once (i.e. adults), we set the survival rate of the first age category (fledglings and individuals younger than 4 years) as equal to 1 in all models. To select the model with the greatest fit to our mark–recapture data, we used AIC_c_. We also calculated Akaike weights as estimates of the relative support for each model.

We built one additional model that represented overall variation among years in the survival rate of the different age classes. This ‘reference’ model included the interaction between year and age affecting *φ* and, as in all previous models, an effect of year on *p*. Given that climatic covariates usually cannot account for all the inter-annual variation in demographic parameters (as estimated by our reference model), we used the *F* statistic proposed by Grosbois *et al*. [47] to test whether the particular combination of explanatory variables selected by AIC_c_ accounted for a statistically significant proportion of the observed inter-annual variation in the survival of all the different age categories. In addition, we calculated the fraction of the temporal variation in the survival of all age classes (as estimated again by our reference model) that can be explained by the climatic covariates selected by AIC_c_. For this purpose, we used a measure of effect size based on the proportion of deviance explained by our most supported model (*R*^2^_*Dev* [47]). This is equivalent to a coefficient of determination and quantifies the importance of climatic covariates in promoting temporal variation in *φ*. An *R*^2^_*Dev* > 0.2 indicates that the explanatory variables selected by AIC_c_ can indeed be considered as influential.

## 3. Results

### 3.1. Fledglings' probability of recruiting

**Figure 1.**
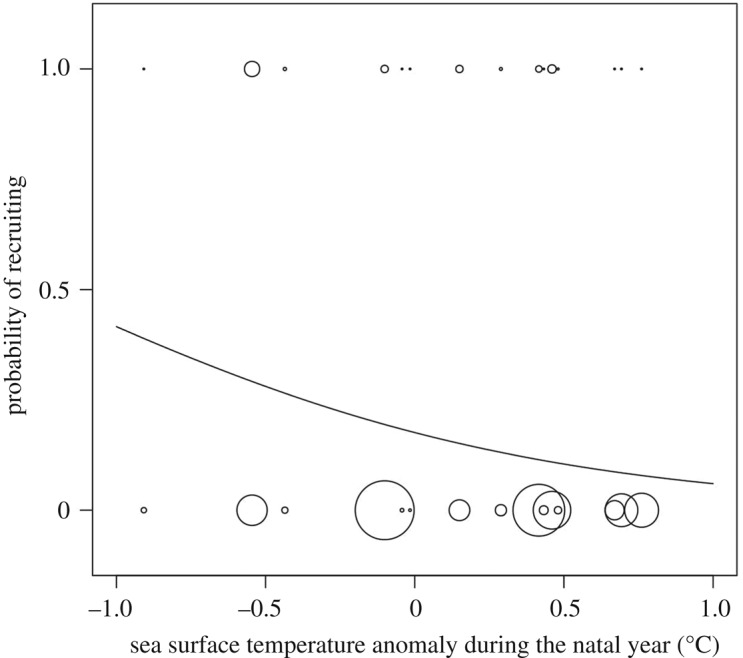
Recruitment probabilities of 8541 fledgling boobies in relation to the annual mean sea surface temperature anomaly they experienced in their natal years (1988–2009). Size of the bubble is proportional to the number of subjects in each category.

The SSTA experienced by fledglings in their natal years predicted their probability of recruiting: the model including both the SSTAn and the number of years of monitoring was the top-ranked model and had 73% of relative support in our data (table 1). Moreover, the model including the SSTAn as the only predictor of recruitment was the second best fitting model and had 21% of relative support, although this model differed by 2.54 AIC_c_ units from the top-ranked model (table 1). The null model and the model including the number of years of monitoring as the only predictor differed by more than 5 and 8 AIC_c_ units from the top-ranked model, respectively, and hence, had weaker support in our data (*w* = 0.05 and 0.01, respectively; table 1). We used the two best fitting models for the model-averaging procedure. The SSTAn was the predictor with the highest relative importance (0.94 versus 0.78 for the number of years of monitoring). The probability of recruiting varied as a negative function of SSTAn (estimate ± s.e.: −1.036 ± 0.435). Each additional degree (°C) of water temperature in the natal year meant a reduction of roughly 50% in fledglings' probability of returning to the colony as adult breeders (figure 1).

**Table 1.** Competing GLMMs with the cohort as a random effect examining the impact of sea surface temperature anomaly during the natal year (SSTAn) on the probability of recruitment of 8541 fledglings of the blue-footed booby (*Sula nebouxii*). Models are listed according to AIC_c_ values, from lowest to highest. Notations: ΔAIC_c_ = difference between any particular model *i* and the top-ranked model; *w* = Akaike weight; *K* = number of parameters.

model	AIC_c_	ΔAIC_c_	*w*	*K*	deviance
recruitment (SSTAn + no. years of monitoring)	6704.7	0.0	0.730	4	6696.7
recruitment (SSTAn)	6707.2	2.54	0.210	3	6701.2
recruitment (no. years of monitoring)	6710.1	5.42	0.050	3	6704.1
null model	6713.1	8.43	0.010	2	6709.1

### 3.2. Adult survival

A single model had strong support in our data (*w* = 0.99; table 2). This top-ranked model included an interaction between age and SSTAc affecting *φ*. The effect of the natal conditions (SSTAn) was included in the second top-ranked model (table 2). However, this second model differed by more than 20 AIC_c_ units from the top model and, hence, had virtually no support (*w* < 0.001). The first model that included an effect of sex on *φ* had no support either (ΔAIC_c_ = 24.7, *w* < 0.001).

**Table 2.** Competing models that examined variation in adult survival (*φ*) of blue-footed boobies (*Sula nebouxii*). Models are listed according to AIC_c_ values, from lowest to highest. In all models, the recapture parameter (*p*) varied among years. SSTAc = sea surface temperature anomaly during the current year; SSTAn = sea surface temperature anomaly during the natal year; *w* = Akaike weight; *K* = number of parameters.

model	AIC_c_	ΔAIC_c_	*w*	*K*	deviance
*φ*(age × SSTAc)	9279.5	0.0	0.99	53	9171.8
*φ*(age × SSTAn)	9299.6	20.1	<0.001	53	9191.8
*φ*(age)	9302.2	22.7	<0.001	39	9223.2
*φ*(age + SSTAn)	9303.8	24.4	<0.001	40	9222.9
*φ*(age + SSTAc)	9303.9	24.4	<0.001	40	9222.9
*φ*(sex + age)	9304.2	24.7	<0.001	40	9223.2
*φ*(age + SSTAc + SSTAn)	9304.3	24.8	<0.001	41	9221.2
*φ*(sex + age + SSTAn)	9305.9	26.4	<0.001	41	9222.9
*φ*(sex + age + SSTAc)	9306.0	26.5	<0.001	41	9222.9
*φ*(sex + age + SSTAc + SSTAn)	9306.3	26.8	<0.001	42	9221.2
*φ*(sex × age)	9317.1	37.6	<0.001	53	9209.4
*φ*(sex × age × SSTAc)	9317.3	37.8	<0.001	81	9151.2
*φ*(age × SSTAc × SSTAn)	9318.6	39.2	<0.001	81	9152.6
*φ*(sex × age × SSTAn)	9341.8	62.3	<0.001	81	9175.7
*φ*(sex × age × SSTAc × SSTAn)	9396.9	117.4	<0.001	137	9111.2
*φ*(SSTAc × SSTAn)	9437.6	158.1	<0.001	29	9379.1
*φ*(sex × SSTAc × SSTAn)	9443.8	164.3	<0.001	33	9377.2
*φ*(SSTAc)	9461.0	181.5	<0.001	27	9406.5
*φ*(SSTAc + SSTAn)	9462.5	183.0	<0.001	28	9406.0
*φ*(sex + SSTAc)	9462.7	183.2	<0.001	28	9406.2
*φ*(sex + SSTAc + SSTAn)	9464.2	184.7	<0.001	29	9405.7
*φ*(sex × SSTAc)	9464.3	184.8	<0.001	29	9405.8
*φ*(.) null model	9474.1	194.6	<0.001	26	9421.7
*φ*(sex)	9476.0	196.5	<0.001	27	9421.5
*φ*(SSTAn)	9476.2	196.7	<0.001	27	9421.7
*φ*(sex + SSTAn)	9478.0	198.5	<0.001	28	9421.5
*φ*(sex × SSTAn)	9479.5	200.0	<0.001	29	9420.9
*φ*(age × year) reference model	9562.3	282.8	<0.001	221	9089.0

**Figure 2.**
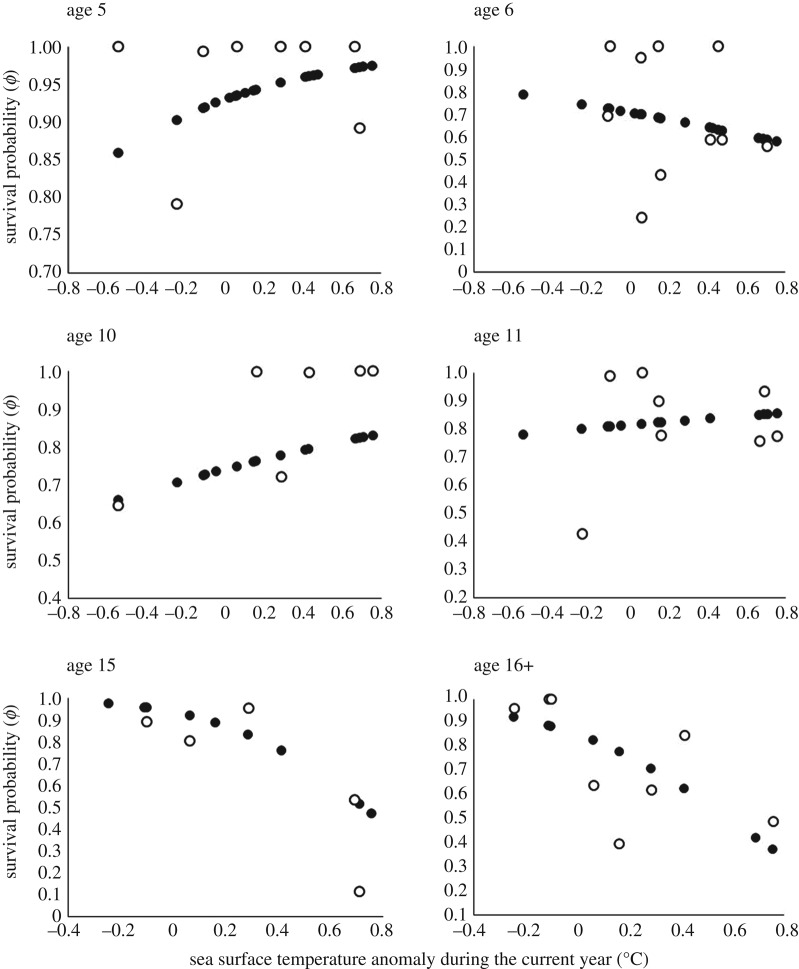
Estimated relationships between annual survival of blue-footed boobies and sea surface temperature anomaly during the current year (SSTAc) at different ages. Black circles depict predicted survival according to the model that best fitted our capture–recapture data (an interaction between age and SSTAc affecting survival). White circles depict estimated survival rates according to our time-varying reference model (an interaction between age and year affecting survival).

Using our dataset, we were able to accurately estimate annual survival rates (estimated from our reference model; electronic supplementary material, table S1) and the expected relationship between SSTAc and survival (estimated from our best fitting model) for six age classes (figure 2). No other parameters were properly estimated because their standard errors were too large. According to our best fitting model, the impact of sea temperature in the current year on annual survival rates of booby adults varied among ages (figure 2). The strongest negative impact occurred in the two oldest age classes. In 15-year-old individuals, survival decreased on average from 0.97 in years when SSTAc was approximately −0.24°C to 0.47 in years when SSTAc was approximately +0.76°C (i.e. a 52% decrease in survival probability). Similarly, in the oldest individuals (≥16 years) survival decreased on average from 0.92 in years when SSTAc was approximately −0.24°C to 0.37 in years when SSTAc was approximately +0.76°C (i.e. a 60% decrease in survival probability; figure 2).

In contrast, younger individuals experienced less drastic changes in survival in response to increases in sea surface temperature (figure 2). According to predictions from our best fitting model, survival of 6-year-old birds decreased on average 26% from the coldest to the warmest year (from 0.78 to 0.58). In 5-, 10- and 11-year-old birds, survival increased on average 13% (from 0.86 to 0.97), 26% (from 0.66 to 0.83) and 9% (from 0.78 to 0.85), respectively, from the coldest to the warmest year (figure 2). This interaction between SSTAc and age was statistically significant (*F*_25,169_ = 20.4, *p* < 0.001) and explained a large amount (75%) of the total inter-annual variation in age-specific survival rates (*R*^2^_*Dev* = 0.75).

## 4. Discussion

This study of the blue-footed booby provides two main results. First, recruitment probability of fledglings declines with increase in sea temperature during their natal years. Second, adult survival over the lifespan of the species is not affected by warm sea temperature during the natal year.

On average, an extra 1.0°C in the natal year meant a reduction of roughly 50% in fledglings' recruiting probabilities. This result may be due to reduced physiological condition of birds born in stressful (warm) years [29,48]. Warming of surface waters associated with El Niño depletes ocean productivity [26,40], with consequent reductions in food quality and quantity for seabirds [27,49]. Boobies born in El Niño years fledge with a low body mass [25,29], and poor body condition at fledging may compromise marine birds' survival and transition into the breeding stage [23,50]. Nonetheless, a number of booby fledglings born in El Niño years manage to recruit despite being underweight, and if so, they recruit earlier and breed less frequently than fledglings born in cool-water years [25]. This probably implies that fledging underweight associated with natal El Niño conditions is still affecting boobies at age 2–6 years, when they return to the colony to try to breed. Recruiting demands considerable energy [51,52] because it involves sexual maturation, nest territory defence and mate acquisition [53]. Individuals stressed by natal El Niño conditions that manage to transit into the adult stage may be less competitive in these domains and, thus, less likely to pair and lay [2,10].

An earlier analysis of individual life histories showed that boobies born in warm El Niño years recruit at a younger age, space their breeding events more widely (i.e. skip more breeding events [35]) and, during the first 10 years at least, achieve similar annual breeding success and total breeding success to boobies born in cool-water years, despite breeding less frequently than the latter [25]. Our results further reveal that for boobies born in warm years, probability of recruitment is low but annual adult survival is undiminished, even at old ages. Thus, we show that when boobies buffer against harsh natal conditions by making life-history adjustments [25], they pay no survival penalty, although an analysis of lifetime reproductive success is still needed. Lack of effect of El Niño-related stresses in the natal year on adult survival also suggests that low-quality fledglings from warm-water years are winnowed out of the population before obtaining breeding status, and this early disappearance may lead to low heterogeneity in the quality of recruits [19,54].

The impact of El Niño conditions in the current breeding year on adult survival varies among age categories, with oldest adults (≥15 years) being the most affected by warm waters. For them, substantial warming (approx. 0.76°C above the historical mean) results in a reduction of approximately 50–60% in probability of survival. In contrast, survival of younger adults shows minimal effects across the whole range of water temperatures. These age-dependent impacts of current El Niño conditions on adult survival may reflect increasing vulnerability to environmental challenges at more advanced ages ([56,57], but see [57]) due, for example, to a deterioration in foraging performance with age [55]. Nevertheless, further studies in wild populations that estimate to what extent illness or physical decay increase vulnerability to severe weather at advanced ages are still needed [55,57].

Our analyses revealed age-dependent impacts of El Niño on adult survival that differ from those reported by an earlier mark–recapture study based on multi-state modelling [28]. The previous study suggested that current El Niño conditions negatively affect survival of the youngest adults, whereas survival of the oldest boobies is largely independent of variation in seawater temperature [28]. This inconsistency probably arises because the previous study pooled age classes 7–16 years into a single category [28], whereas our current analysis included a greater number of advanced age classes, thus providing a better resolution of survival probabilities at older ages and increasing the probability of detecting an effect of SSTAc on survival of the oldest boobies.

## 5. Conclusion

This study contributes to our still limited knowledge of how early-life environments influence vital rates in nature [10,19,23], and provides important insights into the long-term fitness consequences arising from effects of El Niño on early development. Our results also have conservation implications: although developmental adjustments associated with natal El Niño conditions [25] incur no survival penalty late in life, harsh natal conditions reduce recruitment prospects of developing offspring, which may entail subsequent reductions in population growth [14,19,26,31].

## Supplementary Material

Supporting Information figure S1

## Supplementary Material

Supporting Information table S1

## Data accessibility

All relevant data are available from the Dryad Digital Repository: (https://doi.org/10.5061/dryad.9t3js) [58].
