# Supplementary material for: Experiencing El Niño conditions during early life reduces recruiting probabilities but not adult survival
Source: R Soc Open Sci. 2018 Jan 17;5(1):170076. doi: 10.1098/rsos.170076 (PMC5792865; doi:10.1098/rsos.170076)
Supplement: Supporting Information figure S1 [file rsos170076supp1.pdf]

Figure S1

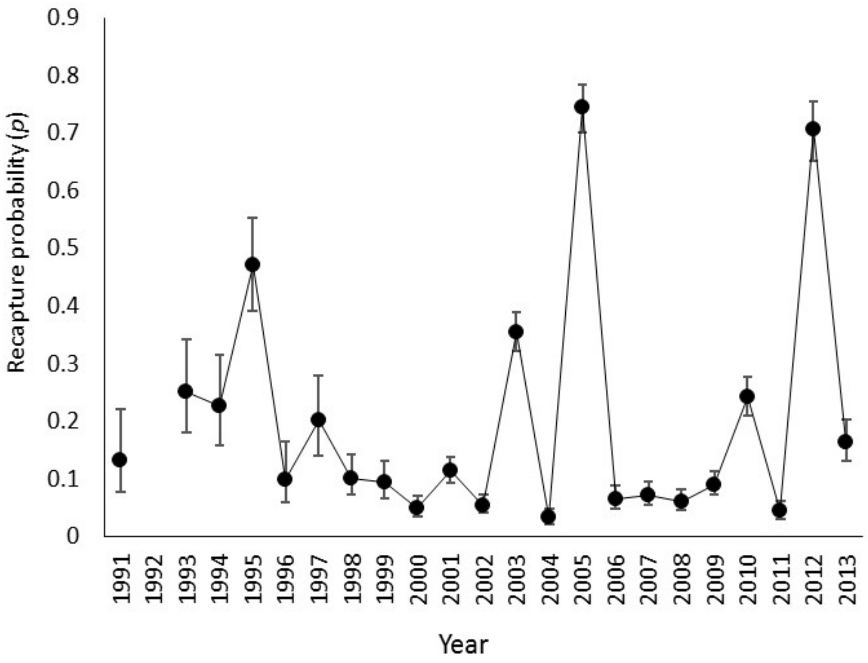

Figure S1. Estimated annual recapture rates from our best-fitting model for adults of one breeding colony of blue-footed boobies (*Sula nebouxii*). Error bars represent 95% confidence intervals. We were not able to accurately estimate recapture rate for 1992.
