# Supplementary material for: Experiencing El Niño conditions during early life reduces recruiting probabilities but not adult survival
Source: R Soc Open Sci. 2018 Jan 17;5(1):170076. doi: 10.1098/rsos.170076 (PMC5792865; doi:10.1098/rsos.170076)
Supplement: Supporting Information table S1 [file rsos170076supp2.pdf]

**Table S1.** Estimated annual survival rates from our reference (time-varying) model for adults of one breeding colony of blue-footed boobies (*Sula nebouxii*). Individuals were classified into different age classes. Standard errors are shown within parentheses. Missing values were either not properly estimated or not biologically possible (e.g., no 10-year-old ringed individuals existed in the colony during the fifth year of our study).

| Annual interval | Age class | | | | | | | | | | | | |
|---|---|---|---|---|---|---|---|---|---|---|---|---|---|
| | 4 | 5 | 6 | 7 | 8 | 9 | 10 | 11 | 12 | 13 | 14 | 15 | ≥16 |
| 1994 – 1995 | -- | -- | 0.58 (0.135) | -- | -- | -- | -- | -- | -- | -- | -- | -- | -- |
| 1995 – 1996 | 0.85 (0.518) | -- | 0.95 (0.134) | -- | -- | -- | -- | -- | -- | -- | -- | -- | -- |
| 1996 – 1997 | -- | -- | -- | -- | -- | -- | -- | -- | -- | -- | -- | -- | -- |
| 1997 – 1998 | -- | -- | 0.99 (<0.001) | -- | -- | -- | -- | -- | -- | -- | -- | -- | -- |
| 1998 – 1999 | 0.99 (<0.001) | -- | -- | 0.62 (0.368) | -- | -- | 0.99 (0.189) | -- | -- | -- | -- | -- | -- |
| 1999 – 2000 | -- | 0.99 (0.001) | -- | -- | -- | -- | 0.64 (0.323) | -- | -- | -- | -- | -- | -- |
| 2000 – 2001 | -- | -- | 0.99 (<0.001) | -- | -- | 0.99 (<0.001) | -- | 0.90 (0.491) | 0.99 (0.008) | -- | -- | -- | -- |

| | | | | | | | | | | | | | |
|---|---|---|---|---|---|---|---|---|---|---|---|---|---|
| 2001 – 2002 | 0.99 (<0.001) | - | -- | 0.86 (0.412) | -- | -- | -- | -- | -- | 0.74 (0.360) | -- | -- | -- |
| 2002 – 2003 | -- | 0.99 (<0.001) | -- | -- | 0.80 (0.412) | -- | -- | 0.76 (0.455) | -- | 0.76 (0.255) | 0.99 (0.094) | -- | -- |
| 2003 – 2004 | 0.99 (0.001) | -- | 0.56 (0.043) | -- | -- | 0.53 (0.144) | -- | -- | 0.66 (0.368) | -- | -- | 0.12 (0.130) | -- |
| 2004 – 2005 | -- | 0.89 (0.031) | -- | 0.99 (<0.001) | -- | -- | 0.99 (<0.001) | 0.93 (0.080) | -- | -- | -- | 0.53 (0.147) | -- |
| 2005 – 2006 | -- | 0.99 (0.229) | 0.69 (0.064) | 0.99 (0.426) | 0.99 (0.001) | -- | -- | -- | -- | -- | -- | -- | 0.99 (0.021) |
| 2006 – 2007 | 0.99 (0.001) | 0.99 (<0.001) | 0.59 (0.179) | -- | 0.99 (0.002) | 0.99 (0.001) | -- | -- | 0.99 (0.794) | -- | -- | -- | 0.85 (0.580) |
| 2007 – 2008 | -- | 0.99 (<0.001) | -- | -- | -- | 0.90 (0.538) | 0.72 (0.108) | -- | -- | 0.34 (0.330) | -- | 0.95 (0.134) | 0.62 (0.619) |
| 2008 – 2009 | -- | -- | 0.99 (<0.001) | 0.77 (0.645) | 0.93 (0.215) | -- | -- | 0.99 (<0.001) | -- | -- | -- | 0.89 (0.001) | 0.99 (0.026) |
| 2009 – 2010 | -- | -- | -- | 0.35 (0.210) | 0.96 (0.638) | -- | 0.99 (0.001) | 0.77 (0.432) | 0.99 (0.001) | -- | -- | -- | 0.49 (0.486) |
| 2010 – 2011 | -- | -- | 0.43 (0.132) | -- | 0.54 (0.367) | 0.34 (0.179) | 0.99 (<0.001) | 0.78 (0.283) | 0.75 (0.308) | 0.99 (<0.001) | -- | -- | 0.39 (0.337) |
| 2011 – 2012 | -- | 0.79 (0.060) | -- | -- | -- | -- | -- | 0.43 (0.086) | 0.32 (0.124) | -- | 0.38 (0.085) | -- | 0.96 (0.596) |
| 2012 – 2013 | 0.57 (0.142) | 0.99 (0.002) | 0.24 (0.097) | -- | 0.89 (0.575) | -- | -- | 0.99 (0.023) | 0.51 (0.234) | 0.67 (0.234) | 0.99 (0.024) | 0.80 (0.424) | 0.64 (0.001) |
